# Clofazimine: A Promising Inhibitor of Rabies Virus

**DOI:** 10.3389/fphar.2021.598241

**Published:** 2021-03-18

**Authors:** Jiajing Wu, Shouchun Cao, Shan Lei, Qiang Liu, Yinghong Li, Yueyang Yu, Hui Xie, Qianqian Li, Xiaoqiang Zhao, Ruifeng Chen, Weijin Huang, Xinyue Xiao, Yongxin Yu, Danqing Song, Yuhua Li, Youchun Wang

**Affiliations:** ^1^ Department of HIV/AIDS and Sex-transmitted Virus Vaccines, National Institutes for Food and Drug Control, Beijing, China; ^2^ Wuhan Institute of Biological Products, Hubei, China; ^3^ Department of Arboviral Vaccine, National Institutes for Food and Drug Control, Beijing, China; ^4^ Beijing Key Laboratory of Antimicrobial Agents, Institute of Medicinal Biotechnology, Chinese Academy of Medical Science and Peking Union Medical College, Beijing, China; ^5^ Institute for Reference Standards and Standardization, National Institutes for Food and Drug Control, Beijing, China

**Keywords:** rabies virus, approved drugs, high-throughput screening, clofazimine, *in vivo*

## Abstract

With an almost 100% mortality rate, rabies virus (RABV) infection is a global concern. Limited post-exposure prophylaxis and lack of an effective treatment necessitate novel antiviral therapies against RABV. Here, using a high-throughput screening (HTS) method developed in our lab, 11 candidates with anti-RABV activity were identified from a library of 767 clinical drugs. Clofazimine (CFZ), an anti-leprosy drug, displayed an EC_50_ of 2.28 μM, and SI over 967 against RABV. Investigations into the underlying mechanisms revealed that CFZ targeted viral membrane fusion at the early stages of virus replication. Moreover, CFZ and Clofazimine salicylates (CFZS) exhibited elevated survival rates *in vivo*, compared with the positive control T-705. Thus, this study revealed CFZ as a promising drug against RABV infection.

## Introduction

Rabies is a viral disease that causes inflammation of the brain in humans and other mammals (Dharmalingam and Jothi, 2015). Rabies virus (RABV), which causes rabies, is a negative-sense single stranded RNA virus of the genus *Lyssavirus*. Rabies can be prevented after a recognized exposure through appropriate wound care and application of post-exposure prophylaxis (PEP) as soon as possible. To prevent rabies virus from gaining access to the nervous system, the World Health Organization (WHO) recommends administering rabies immunoglobulin (RIG) along with four or five doses of rabies vaccine, especially in cases with severe exposure. However, approximately 40,000–70,000 human die annually due to rabies worldwide, roughly 40% of which are children (Davis et al., 2015). The majority of these deaths occurred in Asia and Africa (Fooks et al., 2017). The main reasons are scarcity of RIG and failure to implement appropriate PEP (Jackson, 2013; World Health Organization, 2013).

So far, no effective treatment has been developed to cure the disease after the onset of symptoms (Fooks et al., 2014). Many small molecules have been identified as potential anti-RABV inhibitors but with little promise. Ribavirin, a guanine nucleoside analog, can effectively inhibit RABV replication *in vitro*; however, it lacks clinical efficacy (Anindita et al., 2018). Favipiravir (6-fluoro-3-hydroxy-2-pyrazinecarboxamide), a pyrazine derivative, commonly known as T-705, is a broad-spectrum RNA polymerase inhibitor that has an antiviral effect against RABV *in vitro* (EC_50_ = 32.4 μM, against a RABV vaccine strain) (Arias et al., 2014; Virojanapirom et al., 2016). In mouse models of rabies infection, T-705 (300 mg/kg/day) provided protection when administered 1 h after viral infection (Yamada et al., 2016), but no protection when treatment started 2 days after viral inoculation (Yamada et al., 2019). Higher doses (600 or 900 mg/kg/day) were reported to suppress viral replication in the CNS even when administration started 2 days after inoculation (Yamada et al., 2019). Other studies reported poor efficacy of T-705 *in vivo*, even when T-705 was combined with other anti-viral compounds (Banyard et al., 2019; Marosi et al., 2019). The deficient clinical efficacy suggested that it is not an ideal anti-RABV agent. In 2005, a 15-year-old girl in Wisconsin received a combination treatment with ketamine, ribavirin, amantadine, midazolam, and phenobarbital which later became popularly known as the “Milwaukee Protocol” (Willoughby Jr et al., 2005). Although this girl and four other patients survived after the treatment, at least six others infected with rabies died in the similar treatments. The subsequent failures caused a re-evaluation of the protocol, and it was revealed that ribavirin and amantadine failed to demonstrate efficacy in laboratory animals and recent evidence in primary neuron cultures and mice did not support the use of ketamine (Weli et al., 2006).

Thus, there is an urgent need to discover and develop new antiviral agents against RABV. Instead of developing new drugs, which usually takes many years for the development and has several manufacturing requirements, repurposing of existing drugs is an attractive alternative. The approved drugs are advantageous because their pharmacology and toxicity profiles are known, safety data in humans is established, and manufacturing and formulation feasibility has been demonstrated. In this study, we established a high-throughput screening (HTS) assay based on a pseudovirus containing a firefly luciferase reporter gene enveloped by the RABV glycoprotein G (pRABV) to screen a library of 767 approved drugs for RABV entry inhibitors to identify an effective anti-RABV candidates.

## Materials and Methods

### Cell Lines, Plasmids, Pseudovirus, and Authentic Virus

293T cells (CRL3216, ATCC, Manassas, VA), BSR (variant strain of BHK) cells, PG-4 cells (CRL2032, ATCC) were cultured in 5% CO2 at 37°C in high glucose Dulbecco’s Modified Eagle’s Medium (HyClone, South Logan, UT) supplemented with 10% FBS (Gibco, Carlsbad, CA), and 1% penicillin–streptomycin solution (Gibco). Cells were passaged every 2 days. Production and titration of pseudovirus pRABV and pVSV (an HIV backbone but expresses a vesicular stomatitis virus envelope glycoprotein) were as previously described12. The reason why pVSV was used as a control was that compounds inhibiting HIV replication or luciferase activity will also inhibit pRABV. Virulent rabies virus CVS strain was adapted by BSR cells and BALB/c mice, respectively, and stored at −70°C.

### Compound Library

The 767 compounds, obtained from the National Standard Chemical Control Library of NIFDC were verified to be >95% purity by HPLC analysis. Each compound is currently approved and marketed (as a prescription or over-the-counter medication) in China, and can be administered orally or parenterally. Each compound used in HTS was dissolved in 100% DMSO at a concentration of 30 mM and stored at −20°C.

### Screening Assay With Pseudovirus

Screening was performed in 96-well plates. Eighty wells of each plate were used for test compounds, leaving the first and second columns empty for 0% inhibition (DMSO only, maximum signals = positive control) and background controls, respectively. For primary screening, a stock (30 mM) of each compound was diluted by adding 1 μL of the sample to 99 μL of growth media. Next, the compounds were serially diluted and incubated with 50 μL of pseudovirions pRABV at 37°C, 5% CO_2_ for 1 h. Then the 293T cells were added in 96-well plates at a density of 5 × 10^4^ cells/well and incubated for 48 h at 37°C, 5% CO_2_. After incubation, 150 μL of culture medium per well was discarded and 100 μL of Bright-Glo luciferase reagent (Promega, Madison, WI) was added to each well and reacted with cells for 2 min at room temperature. Finally, 150 μL of lysate per well was transferred to a solid black 96-well plate, and the luminescence signal was collected using a Glomax 96 microplate luminometer (Promega). Percent inhibition was calculated as 100 × [1 - (RLU in the presence of compound - RLU of negative control)/(RLU of positive control-RLU of negative control)].

### Cytotoxicity Testing

The 50% cytotoxic concentration (CC_50_) of drugs were determined by CellTiter Glo luminescent cell viability assay kit (Promega, Madison, WI). Specifically, serial dilutions of drugs, starting from 200 µM were mixed with 293T cells in 96-well plates, then added 50 μL complete medium instead of virus. After incubation at 37°C for 24 h, the cell viability was analyzed using a microplate luminometer (Promega, Madison, WI). The CC_50_ was determined by the dose-response curve using nonlinear regression.

### Rapid Fluorescent Focus Inhibition Test (RFFIT)

Immunogenicity determination of rabies vaccines or natural infection-elicited antibody responses against rabies virus is determined using serological assays including the rapid fluorescent focus inhibition test (RFFIT). This method is also used to verify the effect of compounds. A 100-μL serial dilution of each test compound (9 dilutions in a 3-fold stepwise manner) was incubated with 50 μL of RABV CVS (20, 000 FFU/well) in duplicate for 1 h at 37°C. After neutralization, 50 μL of BSR cells (1 × 10^6^/ml) were added into each well, then plates were cultured for 24 h in a 5% CO_2_ incubator at 37°C. Finally, cells were fixed with pre-chilled 80% acetone at 4°C for 30 min and stained with FITC-conjugated anti-rabies N monoclonal antibody (Fujirebio Diagnostics, Malvern, PA) at 37°C for 30 min (Smith et al., 1973). The fluorescent intensity per well was recorded visually under a fluorescence microscope (Olympus, Tokyo, Japan).

### Binding Affinity Assay

The SPR analysis was performed at 25°C using a BIAcore S200 machine with CM5 chips (GE Healthcare). For all the analyses, PBS-P buffer consisting of 10 mM PBS, pH 7.4, 2.7 mM KCl and 137 mM NaCl, 0.05% surfactant P20 and 5% DMSO was used, and all compounds were exchanged to the same buffer in advance *via* gel filtration. The blank channel of the chip was used as the negative control. RABV were immobilized on the chip at about 4,800 response units. CFZ solutions at gradient concentrations of 0, 0.75, 1.25, 2.5, 5, 10, 20, and 40 μM were allowed to flow over the chip surface. After each cycle, the sensor surface was regenerated with 50% DMSO. The binding kinetics was analyzed with the software of BIA evaluation Version 4.1 using a 1:1 Langmuir binding model.

### Time of Addition Assay

The “time-of-addition” experiment was designed as described previously to determine the virus infection stage blocked by CFZ (Wu et al., 2019). BSR cells were seeded in 96-well plates 1 day in advance. (a) Cell receptor antagonism test: at time point −1h, compound CFZ was added at 4°C for 1 h then washed with phosphate-buffered saline (PBS) three times. BSR cells were then infected with authentic rabies CVS strain at 4°C for 1 h then washed with PBS. (b) CVS fusion/entry test: at time point 0 h, both compound CFZ and CVS strain were added at 37°C for 1 h and washed with PBS. (c) and (d) CVS fusion/post-entry and intracellular biosynthesis test: compound CFZ was added after being infected with CVS strain at 4°C or 37°C for 1. The temperature was set to 37°C for all plates at time point +1 h. After incubation for another 23  h, the inhibition rate was analyzed with Promega microplate luminometer as described previously. Wells treated with DMSO only were used as controls. Data was analyzed based on three independent replicates.

### Fusion Assay

The fusion activity of Rabies virus was determined by counting the number of lyzed cells after the cells were infected. Briefly, 293T cells were first cultured into monolayers in a 24-well plate. Each well was transfected with plasmid pCMV-CVS (Nie et al., 2017). After incubation at 37°C and 5% CO_2_ incubator for 24 h, the medium was replaced by fusion medium buffered with HCl to pH 5.0 for 15 min. The numbers of cells with and without membrane fusions in each well were counted under a microscope, and the data was calculated using ImageJ; at least five wells were counted for each virus sample. Results were derived from two independent assays.

### Molecular Modeling Analysis

The 2D structures of the drugs were downloaded from PubChem database by name, or drawn using ChemDraw software. Discovery Studio 4.5 software (Accelrys Software Inc., San Diego, CA, United States) was used to convert each 2D structure to a 3D molecular model, and the “Prepare Ligands” module was used to add hydrogen atoms and perform energy optimization operations.

### General Procedure for the Preparation of CFZ Salts

CFZ (100 mg, 0.21 mmol), salt-forming acid (0.21 mmol) and three drops of acetonitrile were ground in a mortar-pestle for1 h to give of the desired salt. Clofazimine salicylates (CFZS) mp 243–245°C; Clofazimine hydrochloride salt (CFZH) mp 254–256°C; Clofazimine gallic acid salt (CFZG) mp 177–179°C; Clofazimine aspirin salt (CFZA) mp 238–240°C; Clofazimine methanesulfonate (CFZM) mp 253–255°C.

### Animal Experiments

Mice were handled in accordance with institutional (NIFDC, Beijing, China) guidelines for laboratory animal care and use, and the Animal Care and Use Committee at the NIFDC approved the study protocol. CFZ, Clofazimine salts and T-705, the positive control, were dissolved in 0.5% methylcellulose (Sigma-Aldrich, St. Louis, MO) and administered by oral gavage at 300 mg/kg/day based on earlier studies (Yamada et al., 2016; Yamada et al., 2019). Female BALB/c mice (n = 8–10 per group) were intramuscularly (IM) inoculated in the left hind limb with 100 µL of Rabies virus CVS strain (3.8 lg LD_50_/mL). Next, infected mice were orally administered once daily, starting at 1 h, 1 day, 2 days, or 4 days after infection. Animals were monitored and weighed for up to 14 days post-challenge or until death. Mice were considered to be sick when clinical signs, such as significant weight loss (a 2 g reduction from the day before), secretion around eyes, a foot slip on a stainless-steel wire top clip of a mouse cage, and/or paralyses, were observed.

### Statistical Analysis

GraphPad Prism 6 (San Diego, CA) was used to determine EC_50_ and CC_50_ values of compounds. Statistical significance of percent survival was determined by log-rank (Mantel-Cox) and χ(Davis et al., 2015) tests. *p* values of <0.05 were considered statistically significant.

## Results

### High-Throughput Assay Using Pseudotyped RABV Virus and Authentic RABV CVS Strain

**FIGURE 1:**
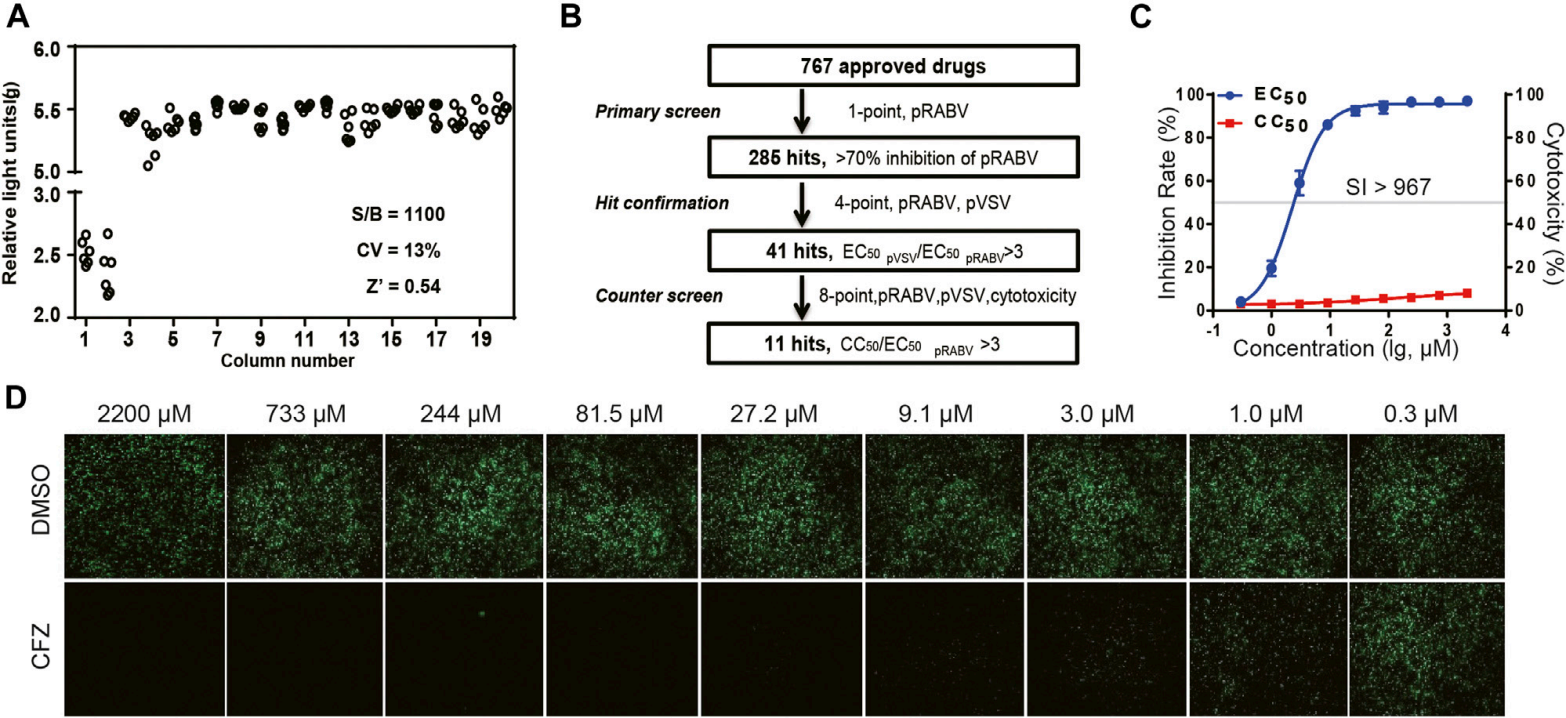
HTS for inhibitors of RABV from 767 compounds. **(A)** Scatter plot of results from DMSO plate screening. Wells in columns 1 and 2 of the 96-well assay plates contained 293Tcells as a control (0% response), whereas pseudotyped RABV (pRABV) was added to all other wells (positive control, 100% response). The signal-to-basal ratio (S/B) in this plate was 1100-fold, with a CV of 13% and Z′ factor of 0.54. **(B)** HTS assay flow chart using pRABV. **(C)**. Inhibition rate of CFZin the RFFIT assay is shown in blue, and cytotoxicity of compounds on host cells is shown in red. **(D)**.The RFFIT assay of CFZ.

Pseudovirus containing a firefly luciferase reporter gene enveloped by the RABV glycoprotein G (pRABV) was used for HTS to select inhibitors of viral entry. HTS conditions including cell-seeding density and pRABV dose were optimized as 50,000 cells/well and 1500 TCID_50_/well, respectively. Under these conditions, the signal-to-basal (S/B) ratio, coefficient of variation (CV) value, and Z’ factor were 1,100, 13%, and 0.54, respectively (Figure 1A), indicating that the experimental requirements are met.

To search for potent inhibitors against RABV, we screened a chemical library composed of 767 approved drugs, as shown in Figure 1B. The primary screen at a single dosage (200 µM) indicated that 285 hit compounds had >70% inhibition, thus necessitating a second round of confirmation. As compounds inhibiting HIV replication or luciferase activity will also inhibit pRABV, pVSV with the same HIV backbone but expressing a vesicular stomatitis virus envelope glycoprotein was used to carry out the second round of screening in parallel. Each of the 285 compounds was diluted at a 1:3 ratio to generate four concentrations ranging from 200 to 7.4 µM to determine their EC_50_ values against pRABV and pVSV. When EC_50 pVSV_/EC_50 pRABV_ ratio was >3, the compound was identified as positive. Thus, 41 compounds were confirmed to block pRABV-mediated infection specifically with little to no effect on pVSV infectivity. Meanwhile, the cytotoxicity of 41 compounds was also detected, and the ratio of CC_50_ and EC_50 pRABV_ was calculated as a selectivity index (SI). After final screening, 11 compounds were confirmed to possess good efficacy against pRABV and exhibited low cytotoxicity, with SI value of above 3 (Table 1).

**TABLE 1:** Anti-RABV activity of 11 hit compounds.

Compounds	CC_50_ (μM)^a^	pRABV EC_50_ (μM)	SI _pRABV_	pVSV EC_50_ (μM)	EC_50_pVSV/EC_50_pRABV	CVS EC_50_ (μM)	SI_CVS_	Approved indication	Mode of action
Clofazimine	>200	1.7	>120	10.5	6.3	2.28	>87.9	Antileprotic	DNA replication inhibitor
Pizotifen	100.5	3.1	32.3	25.7	8.2	67.0	1.5	Antimigraine	Serotonin antagonist
Amlodipine besylate	36.3	1.4	26.8	26.4	19.4	37.4	1.0	Antihypertensive	Calcium channel blocker
Amodiaquine hydrochloride	84.9	4.2	20.3	24.3	5.8	14.8	5.7	Antimalarial	N-desethylamodiaquine activator
Ketotifen fumarate	>200	10.8	>18	87.8	8.1	67.0	>3.0	Antiallergic	H1-antihistamine stabilizer
Amlodipine maleate	34.9	1.9	17.9	25.8	13.3	37.8	0.9	Antihypertensive	Calcium channel blocker
Maprotiline hydrochloride	52	6.9	7.5	32.5	4.7	44.2	>4.5	Antidepressant	H1 receptor antagonist
Irinotecan hydrochloride	>200	33.6	>5.9	>200	>5.9	67.0	1.2	Anticancer	Topoisomerase I inhibitor
Dioxopromethazine hydrochloride	>200	36.7	>5.5	>200	>5.5	67.0	>3.0	Antipsychotic	Dopamine receptor antagonist
Cisplatin	>200	39.1	>5.1	>200	>5.1	67.0	>3.0	Anticancer	DNA replication inhibitor
Tetracaine hydrochloride	>200	65.8	>3	>200	>3	67.0	>3.0	Anesthetic	Calcium channel blocker

A highest concentration of inhibitors evaluated for cytotoxicity assays was 200 μM.

To verify results obtained from HTS, we next examined the antiviral activity of 11 hits with an authentic RABV CVS strain using the RFFIT method (Table 1). The compounds were diluted at a 1:3 ratio to generate nine concentrations ranging from 2,200 to 0.3 µM. Clofazimine (CFZ) was found to be efficacious against CVS with an EC_50_ value of 2.28 μM, and a low level of cytotoxicity (SI of >967) (Figure 1 C and D). CFZ is a riminophenazine that used for the treatment of leprosy and *tuberculosis* (Lu et al., 2011; Cholo et al., 2012; Liu et al., 2012; Murashov et al., 2018).

### Binding Affinity Assay and Time-Of-Addition Effect of CFZ on RABV Life Cycle

**FIGURE 2:**
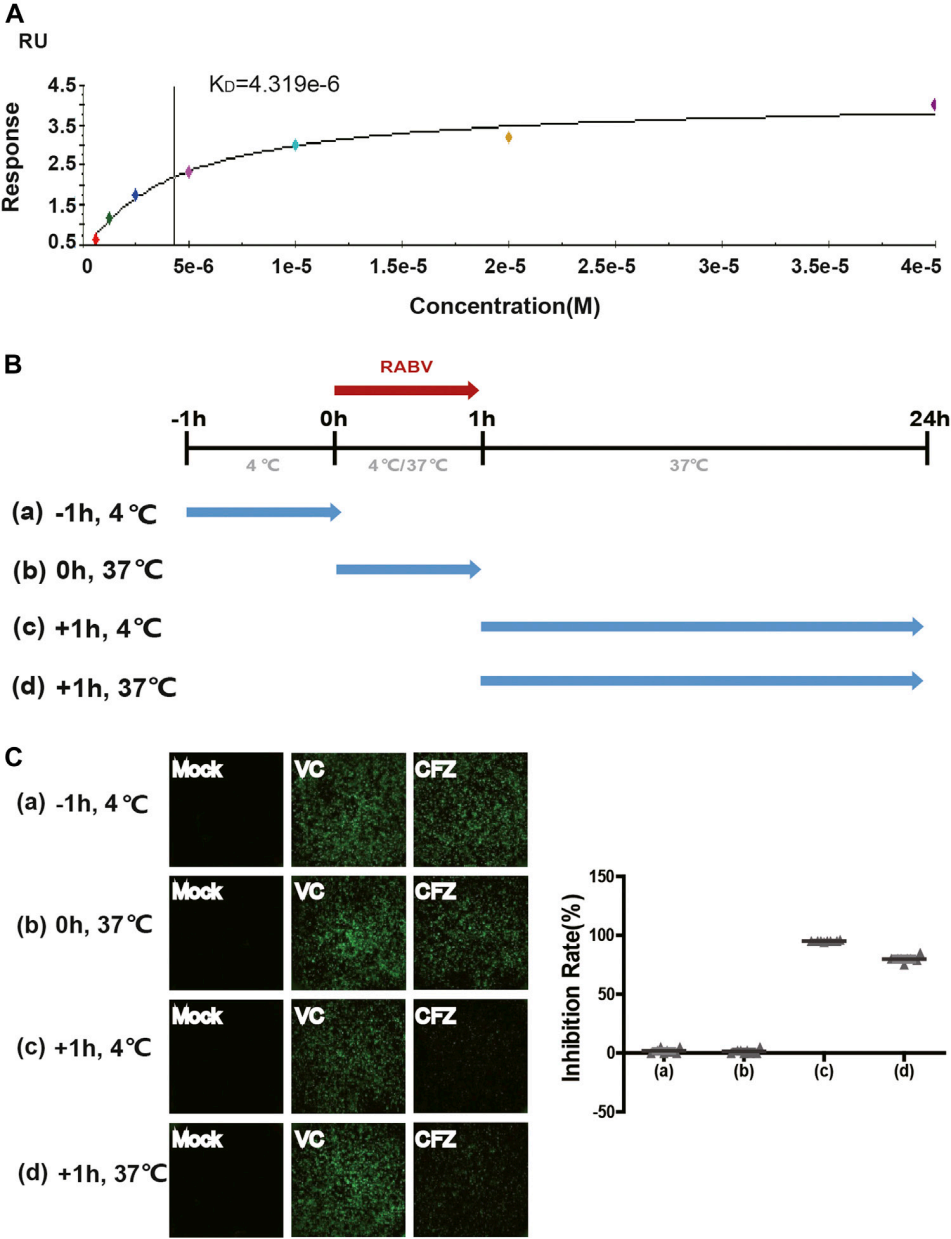
Anti-RABV mechanisms of CFZ. **(A)** Binding of RABV and CFZ by BIAcore assay. **(B)** Schematic diagram of the “time-of-addition”. Blue arrows show the period when CFZ was present. Red arrows show the period when RABV CVS strain was present. **(C)** Inhibition of RABV infection was detected as a decrease in fluorescence activity. Inhibition rates of RABV infection were presented on the right.

To better understand how CFZ acted on RABV, the binding affinity and kinetics of CFZ was determined by SPR at 25°C. Under the conditions tested, the binding affinity (K_D_) of CFZ to RABV was 4.319 μM (Figure 2A). These initial results suggested a possible link between CFZ and RABV. Furthermore, the “time-of-addition” experiment was performed to elucidate which step of viral replication was inhibited by CFZ (Figure 2B). CFZ (25 µM) was added to BSR cells before authentic RABV CVS strain infection (-1 h), during infection (0 h), and 1 h post infection (+1 h) and incubated for 1 h. Control wells were treated with compound vehicle (DMSO). All assays were performed in octuple. As shown in Figure 2C, no suppression of fluorescence activity by CFZ was observed at −1 h or 0 h point, suggesting that CFZ did not inhibit RABV infection either by directly binding to RABV or blocking the cell receptors. However, CFZ exerted a strong inhibitory effect when added 1 h post infection both at 4 and 37°C, suggesting that CFZ worked on viral membrane fusion and genome replication.

### CFZ Inhibits G-Mediated Membrane Fusion

**FIGURE 3:**
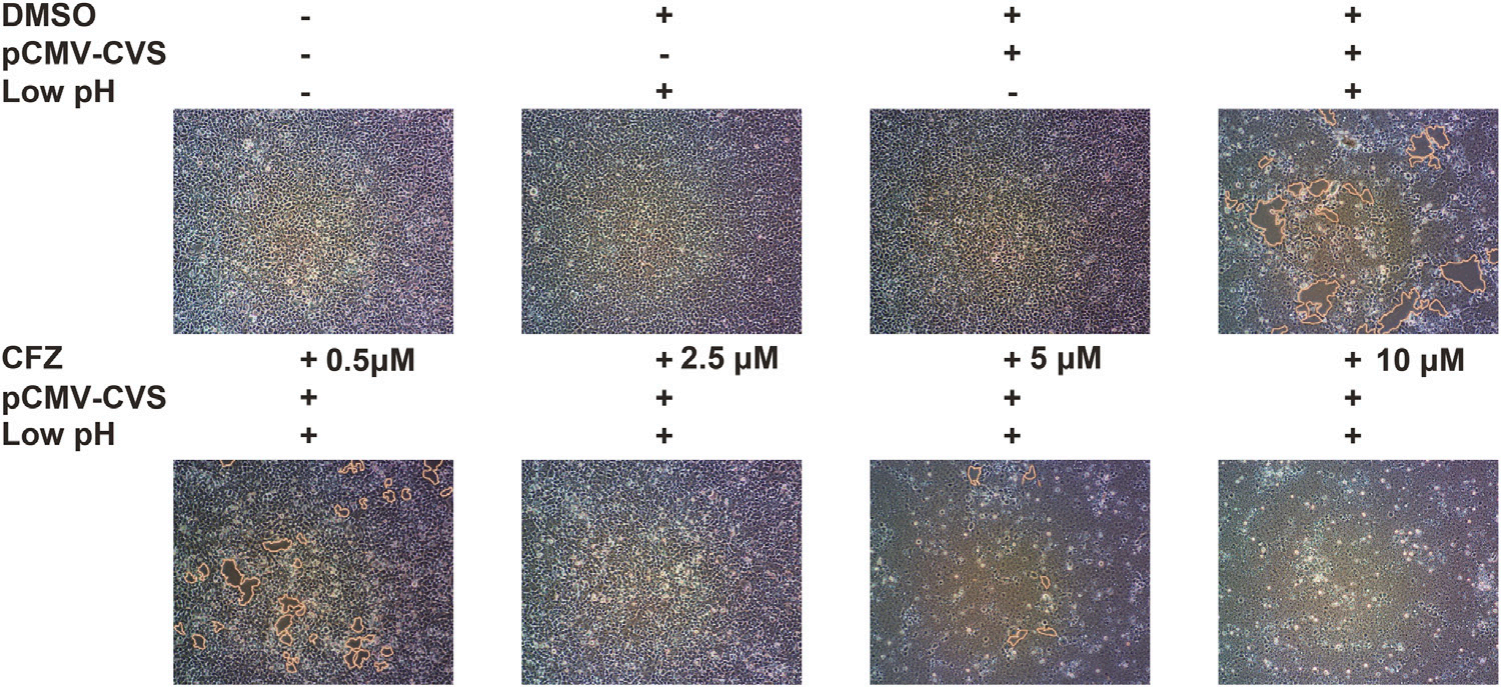
CFZ inhibited G-mediated membrane fusion. 293T cells were transfected with pCMV-CVS. After 24 h, CFZ or DMSO were added for 1 h followed by treatment with fusion medium buffered (pH 5.0) for 15 min. Then, the buffer was replaced with neutral pH DMSO with 10% FBS. Untransfected cells were used as controls. The cells with membrane fusions were outlined with yellow lines. The data were detected with ImageJ and were performed as three independent experiments.

G-mediated membrane fusion is a common condition which has considerable impact on a low-pH. In order to confirm whether CFZ could inhibit RABV infection at a membrane fusion step, a glycoprotein G-mediated membrane fusion assay was designed. 293T cells transfected with pCMV-CVS, a plasmid expressing CVS glycoprotein G, were incubated with either compound and subjected to a low-pH pulse to promote fusion. As shown in Figure 3, low pH triggered membrane fusion in pCMV-CVS-transfected cells, whereas a neutral pH showed no effect. CFZ significantly inhibited syncytium formation at concentration of 10 µM. These results indicated that blockade of glycoprotein G-mediated membrane fusion was one mechanism underlying CFZ inhibition of RABV infection. CFZ may also inhibit RABV infection by impacting infected host cells, for example by modulating host potassium channels or by inducing the generation of reactive oxygen species within these cells (Cholo et al., 2012; Leanza et al., 2014; Faouzi et al., 2015).

### Molecular Docking of CFZ With RABV Glycoprotein G

**FIGURE 4:**
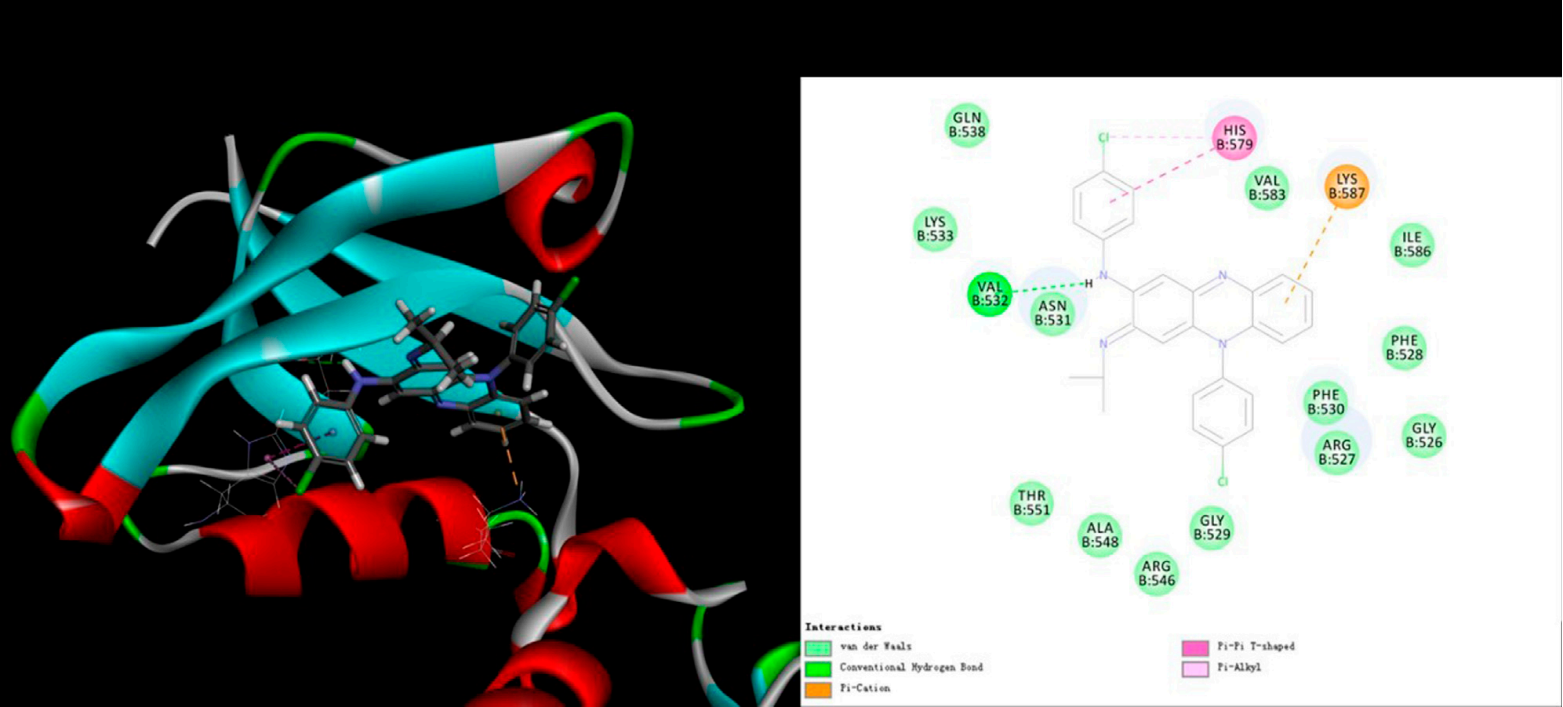
Receptor-ligand interaction model of CFZ with RABV glycoprotein G by CDOCKER calculation. **(Left)** Solid surface map of the interaction pocket with CFZ. Red, blue, and white colored regions correspond to negatively charged, positively charged, and neutral areas, respectively. **(Right)** Ligand is colored by element type (C, yellow; N, blue; Cl, green), key bonds are indicated by dashed lines between the atoms involved, and the colors of key bonds and residues are shown according to the interaction mode (van der Waals, light green; hydrogen bond, green; Pi-cation, orange; Pi-sigma, purple and Pi-alkyl, mauve pink).

To investigate their interaction, LibDock docking (including and CDOCKER analyses) was performed for CFZ and RABV glycoprotein G. The co-crystal structure (Protein Data Bank ID: 3NKF) of PTPN4 PDZ domain complexed with the C-terminus of a rabies virus G protein was selected to be the receptor model. CFZ displayed a reasonable LibDock score of 120.362, indicating a good interaction between CFZ and RABV glycoprotein G. In addition, the interaction binding energy of 33.0582 kcal/mol, indicated direct interaction between CFZ and RABV glycoprotein G. CFZ fitted well in the active binding site of RABV glycoprotein G (Figure 4), and the major interactions included hydrogen bond, van der Waals forces, Pi-cation and hydrophobic interaction, might contribute together to the strong interactions (Figure 4). The docking configuration is merely suggestive and needs extensive experimental validation.

### Anti-RABV Activity of Clofazimine Salts *in vitro* and *in vivo*

**FIGURE 5:**
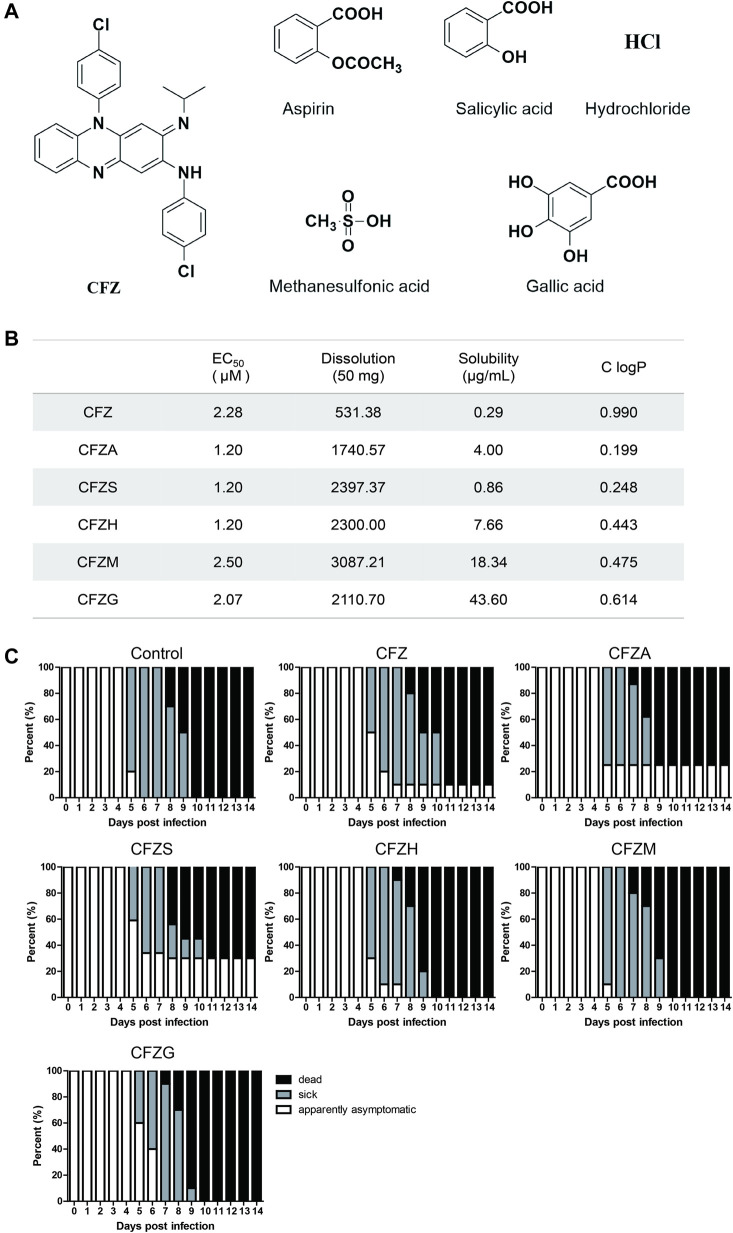
Anti-RABV activity of five Clofazimine salts *in vitro* and *in vivo.* **(A)** Structure of Clofazimine and other five Clofazimine salts. **(B)**. EC50s, dissolutions, solubilities, and ClogPs of Clofazimine and other Clofazimine salts. **(C)**.The survival curves of mice treatment with different compounds. Mice were intramuscularly inoculated with RABV and orally administered CFZ (150 mg/day), CFZA (207 mg/day), CFZS (194 mg/day), CFZH (162 mg/day), CFZM (181 mg/day), and CFZG (204 mg/day), respectively, for 7 days beginning 1 day after inoculation. Mice were monitored for 14 days “Sick” indicates that mice had significant clinical signs. Log-rank test was used for comparison survival curves. The data a presented as three independent experiments.

There were obvious side effects of pigmentation in skin when CFZ was used in the treatment of leprosy, originating from its high lipid solubility. Therefore, five different CFZ salts including CFZA, CFZS, CFZH, CFZM, and CFZG were prepared in equal molar ratio to improve the pharmacokinetic and pharmacodynamic property (Figure 5A). Then, the antiviral activity and cytotoxicity of these salts were examined using CVS, as described previously. The calculated EC_50_ values, solubility, and lipid/water distribution coefficient (ClogP) values for CFZA, CFZS, CFZH, CFZM, and CFZG are shown in Figure 5B. CFZS, CFZA and CFZH were the most potent inhibitors of RABV infection with EC_50_ = 1.2 μM.

Most of these clofazimines salts exhibited higher anti-RABV activity, improved solubility as well as lower lipophilicity on the basis of CFZ, indicating that the salt forming modification of CFZ might be beneficial for the antiviral potency enhancement because of the physicochemical property improvement.

To further confirm antiviral activity, we evaluated the treatment effects of CFZ and CFZ salts in a murine infection model using the CVS strain, which was administered intramuscularly. IM administration allowed a longer incubation period and relatively slow symptom appearance, which was conducive to investigate the preventive effects of treatment after exposure. In this model, the virus enters the brain through peripheral nerves and then proliferates massively in the central nervous system. Infected mice first lose hair luster and then develop flaccid paralysis, which extends from the hind limbs to the whole body. In our study, weight loss and quadriplegia were considered as indices of disease. Balb/c mice due to their small size and convenience to study are more suitable than golden hamsters and beagle dogs for large-scale screening. CFZ (150 mg/kg), CFZA (207 mg/kg), CFZS (194 mg/kg), CFZH (162 mg/kg), CFZM (181 mg/kg), and CFZG (204 mg/kg) were orally administered 1 h after IM inoculation of virus. Mice were treated once daily for 7 days and monitored for a total of 14 days after virus exposure.

As shown in Figure 5C, untreated BALB/c mice were observed to be sick and they died on the fifth and eighth day post infection (dpi), respectively. Overall, the mortality was 100% for the control group, and all of the sick mice were dead by 10 dpi. CFZ, CFZA, and CFZS improved the overall survival rates of mice infected with CVS compared with controls; there was still a tendency of CFZ, CFZS, and CFZG to delay the onset time of symptoms and prolong survival. At five dpi, percentages of sick mice in control, CFZ, CFZS, and CFZG treatment groups were 80, 50, 40, and 40%, respectively. Moreover, at 14 dpi, survival rates in CFZ, CFZA, and CFZS treatment groups were, 10, 25, and 30%, respectively. In contrast, CFZH, CFZM, CFZG produced no improvement on the survival rate compared with the control. No redness in the skin was observed in mice treatment with clofazimine salts compared with CFZ. Although the physicochemical properties of CFZS, the most efficacious compound, predict higher permeability of the blood-brain barrier and lower accumulation in adipose tissue, future pharmacokinetic studies will have to be performed to verify these predictions.

### CFZS Exhibited Superior anti-RABV Activity Than T-705 When Administered one or 2 days Post-challenge

Given the encouraging effect of CFZS, we designed an additional experiment to evaluate the antiviral efficacy of CFZ and CFZS upon initiation of administration at different time points post-exposure. Favipiravir (also known as T-705) was used as the positive control because it was efficacious in mouse models of RABV infection when administered at 300 mg/kg/day 1 h after viral inoculation (Yamada et al., 2016; Yamada et al., 2019). In this experiment, mice intramuscularly inoculated with RABV CVS strain were orally administered T-705 (300 mg/kg), CFZ (150 mg/kg) and CFZS (194 mg/kg) beginning at different time points. Mice were monitored for 14 days and the Kaplan-Meier survival curves were shown for individual studies with appropriate vehicle controls.

**FIGURE 6:**
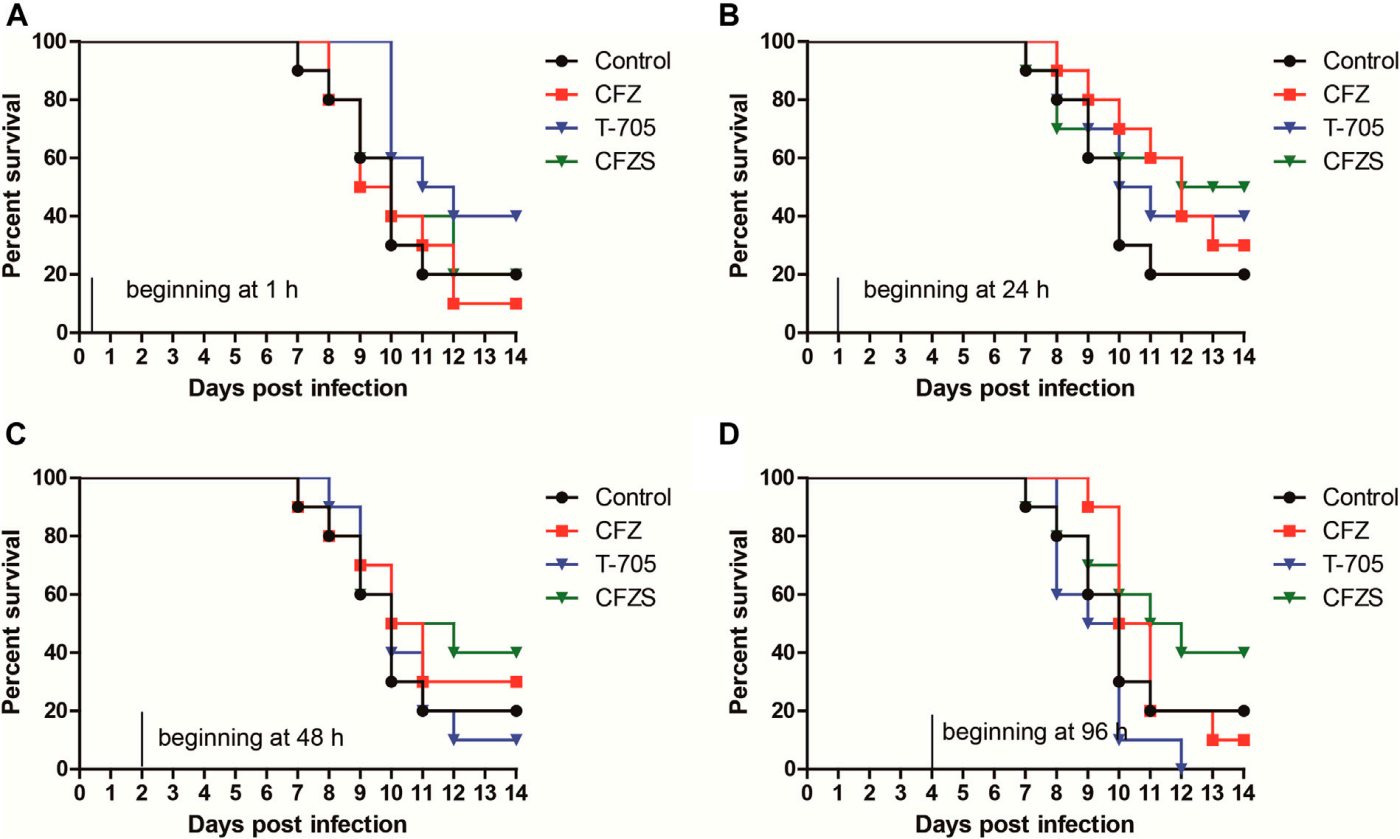
Post-RABV challenge effect of CFZ, CFZS and T-705 on the survival rate of mice. Mice were orally administered CFZ (150 mg/kg/day), CFZS (194 mg/kg/day) or T-705 (300 mg/kg/day) beginning 1 h (0 days) **(A)**, 1 day **(B)**, 2 days **(C)**, or 4 days **(D)** after inoculation and ending at 6 days **(A)** or 7 days **(B**–**D)** after inoculation.

T-705 treatment (300 mg/kg/day) started 1 h or 24 h after RABV CVS infection resulted in 40% survival at 14 dpi, while 10 and 0% mice survived when treatment was started 48 and 96 h after infection (Figures 6A–D). Our results corroborate earlier reports (Yamada et al., 2016; Yamada et al., 2019). We did not try higher doses of T-705 (600 or 900 mg/kg/day) that had been reported to suppress viral replication in the CNS when treatment was started 48 h after infection (Yamada et al., 2019).

Compared with T-705, CFZ and CFZS showed different results for optimal administration time. CFZ administration beginning at 1 h or 96 h post-challenge both did not show any antiviral efficacy. However, CFZ groups treated beginning at 24 h or 48 h post-challenge exhibited a modest increase in survival. The survival rates of both groups were 30%, which was 10% higher than vehicle control (*p* = 0.2534, and *p* = 0.5326, respectively). CFZS administration beginning at 24, 48 or 96 h post-challenge exhibited superior anti-RABV activity than T-705, with survival rates of 50, 40, and 40%, respectively (*p* = 0.7462, *p* = 0.3258, and *p* = 0.0388, respectively). No redness in the skin was observed in mice treatment with CFZS.

### Anti-RABV Activities of CFZ and CFZS in a Neuronal Cell Line

**FIGURE 7:**
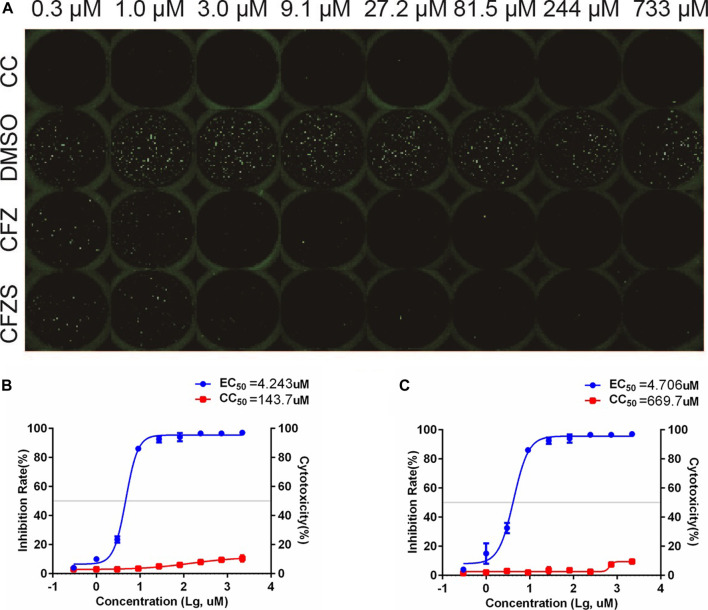
Anti-RABV activities of CFZ and CFZS in PG-4 cells. The RFFIT assay and EC_50_ of CFZ **(A, B)** and CFZS **(A, C)** in PG-4 cells.

Since RABV is a neurotropic virus, it is essential to show that potential therapeutics suppress RABV infection in nervous tissue. We therefore used the cat astrocytoma cell line PG-4 and the *in vitro* RFFIT assay to assess CFS and CFZS. Both compounds showed anti-rabies virus activity in PG-4 cells with EC_50_ values of 4.243 and 4.706μM, respectively (Figure 7).

## Discussion

At present, there are still no effective treatments targeting for rabies patients with clinical symptoms. A few rabies patients were cured by Milwaukee protocol, but the therapy yield positive results in about 40 cases in the following years (Willoughby et al., 2005; Brazilian Rabies Survivor, 2008; Wiedeman, 2012). Importantly, the high cost and continuous cold chain transportation of rabies vaccine and RIG has made the development of RABV-specific inhibitors pertinent. In recent years, researchers have focused on “drug repurposing”, that is, exploring new pharmacological targets for old drugs. Since studies into the tolerance, delivery characteristics and kinetics of these old drugs are already available, the drugs can be used more effectively and safely. Moreover, compared with traditional new drug development, drug repurposing takes less time and cost (Ulferts et al., 2016; Wang et al., 2017). Thus, the scope of this paper lies in screening hundreds of FDA approved compounds for RABV-specific antiviral activity and identifying the specific hits, so that they can be brought to the clinic.

The current assays for immunogenicity determination of rabies vaccines or natural infection-elicited antibody responses against rabies virus are determined using serological assays including the rapid fluorescent focus inhibition test (RFFIT), fluorescent antibody virus neutralization (FAVN) test and enzyme linked immunosorbant assay (ELISA) (Smith et al., 1973). Currently, the “gold standard” for *in vitro* assays are RFFIT and FAVN, both of which are routinely used in WHO reference laboratories. Our group established a RABV-neutralizing antibody assay based on pRABV and compared it with the RFFIT assay to define an optimal method based on criteria of simplicity, speed, and sensitivity (Wu et al., 2019). Here, the pseudovirus system was used to establish a high-throughput assay for anti-RABV drug screening. By incorporating an appropriate reporter gene, pseudotyped viruses were made amenable to HTS assay formats, which saves washing steps, time for fixation, and antibody incubation compared with the RFFIT assay. Moreover, replication-defective pseudotyped viruses allow for testing at lower biological containment facilities, enabling the use of state-of-the-art robotics platforms to assess efficacy of multiple concentrations of each compounds for hit confirmation (Basu et al., 2010).

Based on HTS of 767 compounds, we identified 11 candidates exhibiting antiviral activity against pRABV, and then were confirmed by authentic RABV. Of the 11 candidates, CFZ, the first-line drug for the treatment of leprosy (Browne et al., 1981), had a strong inhibition of RABV activity *in vitro*, with EC_50_ of 2.276 μM, CC_50_ > 2,200 μM, and SI > 967. The results were replicated in PG-4, a cat astrocytoma cell line, to verify the activity of CFZ and CFZS (Figure 7). Generally, the replication of RABV goes through the following stages: adsorption, penetration, shelling, transcription, translation, replication, assembly, and budding release. At a low pH (below 6.2), RABV enters the host cells relying on a glycoprotein G-mediated membrane fusion.

The results of the affinity analysis, time-of-addition assay, and fusion analysis in the present study indicated that CFZ inhibited RABV infection at the fusion step. Our mechanistic studies indicated that blockade of glycoprotein G-mediated membrane fusion was one mechanism underlying CFZ inhibition of RABV infection at the fusion step, although we could not exclude direct effects of CFZ on the infected host cell.

It was worth noting that CFZ had been reported to block potassium channels (Leanza et al., 2014; Faouzi et al., 2015). This blocking of potassium channels in infected cells would change the membrane potential and alter calcium signaling, and could thereby hinder viral membrane fusion and genome replication. Because of its high lipophilicity and redox potential, CFZ was also considered to cause the production of superoxide and hydrogen peroxide (Cholo et al., 2012). In infected cells, this reactive oxygen species may inhibit viral membrane fusion and genome replication. For the neurotropic virus such as RABV, the virus infection is a complex process *in vivo*. These initial steps in characterizing the antiviral activity of CFZ and CFZS should be followed up with additional studies such as identification of the drug target. Further studies are needed to define the mechanisms by which CFZ and CFZS inhibit RABV infection both *in vitro* and *in vivo*.

CFZ, a drug used for the treatment of leprosy and *tuberculosis*, belongs to the class 2 of biopharmaceutical classification system (BCS), which is characterized by low solubility and high permeability (Cholo et al., 2012; Murashov et al., 2018). Although CFZ showed its strong inhibitory activity against RABV *in vitro*, the higher concentration in subcutaneous fat tissue and extremely low content in cerebrospinal fluid may account for the lower anti-RABV activity *in vivo.* Obviously, for the neurotropic virus such as RABV, increasing the concentration of antivirals in central nervous system (CNS) is the key to improve the antiviral activity (Quiñones-Torrelo et al., 2002). Therefore, CFZS was designed as the ideal alternative of CFZ to improve anti-RABV activity *in vivo* with a higher permeability to the blood-brain barrier and a less accumulation in adipose tissue. Pharmacokinetic studies need to be done to determine if CFZS crosses the blood-brain barrier and accumulates to a lesser extent in adipose tissues than CFZ.

To simulate the natural infection mode of human being bitten by dogs infected with RABV, mice were challenged by IM injection. Compared with intracerebral injection, challenging by IM allowed longer incubation period and a relatively slow symptom appearance, which is conducive to investigate the preventive effect of the treatment after exposure. Morever, Further studies on the anti-RABV effects of CFZ and CFZS could be perfomed on large laboratory animals such as Beagles even non-human primates.

In this study, CFZ and CFZS increased survival when administration was begun 1 day or 2 days after infection, but there was no statistical significance between treatment groups and vehicle control group. We found that efficacies of CFZ, CFZS, and T-705 against RABV showed different tendencies when administration was delayed. T-705 was effective when administered beginning 1 h or 1 day after inoculation. However, the CFZ and CFZS treated group exhibited better survival when administration commenced 1 day or 2 days after inoculation. This difference suggested that CFZ and T-705 may act on different stages of RABV infection and/or have different mechanisms of antiviral activity. Therefore, CFZ or CFZS might be used in combination with T-705 to prevent RABV infection under the conditions of unavailable PEP.

Given the high mortality and absence of approved drugs for RABV infections, experimental therapies with limited proof-of concept data for efficacy are worthy of application in infected patients under expanded use authorizations. Ketamine reportedly inhibits RABV replication in cell cultures at high concentrations (1–2 mM) by inhibiting genomic transcription (Lockhart et al., 1992). Compared with ketamine, CFZ can inhibit RABV at a lesser concentrations (2–33 μM). However, the negative results *in vivo* highlight concerns about extrapolating from *in vitro* to *in vivo* and the question of how EC_50_ values from *in vitro* assays correlate to protective levels in animal models. Factors such as the type of formulation (research-grade or pharmaceutical), drug-drug interactions, or even interspecies differences in gastrointestinal adsorption may have effects (Pelkonen et al., 2001). Importantly, for the treatment of neurological diseases, many obstacles must be overcome to effectively deliver therapeutic agents to the central nervous system, such as uptake of the drug by the blood-brain barrier and blood-spinal cord barrier.

## Conclusion

In summary, we have demonstrated for the first time that CFZ and CFZS exhibit potential activity against RABV infection worthy for next investigation. This study lays the foundation for identifying more effective RABV inhibitors, and discovering more optimal analogs for the treatment of RABV infection by modifying the CFZ structures.

## Data Availability

The original contributions presented in the study are included in the article/Supplementary Material, further inquiries can be directed to the corresponding authors.

## Abbreviations

NIFDC, National Institutes for Food and Drug Control; RABV, rabies virus; RFFIT, rapid fluorescent focus inhibition test; HTS, high-throughput screening; PEP, post-exposure prophylaxis; CFZ, Clofazimine; pRABV, pseudotyped rabies virus; pVSV: pseudotyped vesicular stomatitis virus; EC50, 50% effective concentration; CC50, 50% cytotoxic concentration; SI, Selectivity index.
